# Prognostic Value of Metastatic Tumoral Caveolin-1 Expression in Patients with Resected Gastric Cancer

**DOI:** 10.1155/2017/5905173

**Published:** 2017-07-30

**Authors:** Der Sheng Sun, Soon Auck Hong, Hye Sung Won, Su Hyun Yoo, Han Hong Lee, Okran Kim, Yoon Ho Ko

**Affiliations:** ^1^Division of Oncology, Department of Internal Medicine, The Catholic University of Korea, Seoul, Republic of Korea; ^2^Department of Pathology, Soonchunhyang Cheonan Hospital, Soonchunhyang University College of Medicine, Cheonan, Republic of Korea; ^3^Department of Hospital Pathology, Uijeongbu St. Mary's Hospital, College of Medicine, The Catholic University of Korea, Uijeongbu-si, Republic of Korea; ^4^Department of General Surgery, The Catholic University of Korea, Seoul, Republic of Korea; ^5^Cancer Research Institute, College of Medicine, The Catholic University of Korea, Seoul, Republic of Korea

## Abstract

**Objective:**

Caveolin-1 (Cav-1), as the main component of caveolae, has complex roles in tumourigenesis in human malignancies. We investigated Cav-1 in primary and metastatic tumor cells of gastric cancer (GC) and its association with clinical outcomes.

**Methods:**

We retrieved 145 cases of GC who had undergone curative gastrectomy. The expression levels of Cav-1 was evaluated by immunohistochemistry, and its association with clinicopathological parameters and patient survival was analyzed.

**Results:**

High expression of Cav-1 protein of the GC in the stomach and metastatic lymph node was 12.4% (18/145) and 16.5% (15/91). In the multivariate analysis, tumoral Cav-1 protein in metastatic lymph node showed prognostic significance for relapse-free survival (RFS, HR, 3.934; 95% CI, 1.882–8.224; *P* = 0.001) and cancer-specific survival outcome (CSS, HR, 2.681; 95% CI, 1.613–8.623; *P* = 0.002). Among the GCs with metastatic lymph node, it remained as a strong indicator of poor prognosis for RFS (HR, 3.136; 95% CI, 1.444–6.810; *P* = 0.004) and CSS (HR, 2.509; 95% CI, 1.078–5.837; *P* = 0.032).

**Conclusion:**

High expression of tumoral Cav-1 protein in metastatic lymph node is associated with unfavorable prognosis of curative resected GC, indicating the potential of novel prognostic markers.

## 1. Introduction

Gastric cancer (GC) still remains the third leading cause of cancer-related mortality, with 723,100 deaths per year [1]. With recent advancements in our understanding of the molecular biology of GC, targeting agents for molecular targets, such as epidermal growth factor receptor (EGFR), vascular endothelial growth factor (VEGF) receptor, and human epidermal growth factor receptor 2 (HER2), have been used widely to improve patient survival in the setting of recurrent and metastatic GC [2, 3], but the prognosis of patients with advanced GC remains poor. Therefore, new therapeutic molecular targets are required to improve the survival of patients.

Caveolin-1 (Cav-1), a 22-kD protein of 178 amino acids and a member of the caveolin family (Cav-1, 2, and 3), is the highly conserved and essential component of caveolae [4]. Cav-1 is expressed in the terminally differentiated cells, such as fibroblasts, adipocyte, endothelial cells, myoepithelial cells, and type I pneumocytes, but not in human peripheral blood cells or myeloid, lymphoid, and erythroid cell lines. Functionally, Cav-1 has been implicated in diverse cellular processes, including cholesterol homeostasis, vesicular transport, cell migration, cell cycle, and cell polarity, to regulate cell transformation and signal transduction. The perturbations in Cav-1 expression and/or function were, therefore, assumed to play an important part in disease pathogenesis, such as cancer [5, 6].

Dysregulation of Cav-1 has been associated with several human malignancies including GC. Several studies implicate that Cav-1 is involved in a tumor suppression in vitro and in vivo [7, 8]. In contrast, others reported an increased expression of Cav-1 in the more advanced stages of cancers [5, 9, 10], which still suggest the conflicting impact on cancer progression of Cav-1 protein. These contradictory results could be due to the complex biologic behavior of Cav-1 protein, which depends on the location of this molecule and interaction of signaling pathways [11], which might mean the different roles between primary and metastatic tumors. Thus, to clarify the clinical role of the expression of Cav-1 protein in the curatively resected GC, we evaluated the expression of tumoral and stromal Cav-1 proteins in primary gastric tumors and metastatic lymph nodes and compared their relationship with clinicopathological parameters and clinical outcomes.

## 2. Materials and Methods

### 2.1. Patients and Specimens

The clinical and pathological data of patients with gastric cancer, who had undergone primary curative resection between 2001 and 2005 at Uijeongbu St. Mary's Hospital of the Catholic University of Korea, were reviewed. The inclusion criteria were the following: (i) pathologically confirmed diagnosis of adenocarcinoma; (ii) having performed primary R0 resection of cancer and not received radiation or chemotherapy preoperatively; (iii) having at least 15 or more the removed lymph nodes; and (iv) paraffin block of tumor specimens were available. Postoperative pathological staging was analyzed according to the American Joint Committee on Cancer staging criteria, 7th edition. This study was approved by the Institutional Research Ethics Board of Uijeongbu St. Mary's Hospital of the Catholic University of Korea and adhered to the Declaration of Helsinki.

### 2.2. Immunohistochemical Analysis

Immunohistochemistry was performed on formalin-fixed, paraffin-embedded tissues of all primary cancer samples and lymph nodes with cancer metastasis. For immunostaining for Cav-1, we excluded meticulously low tumor volume (<2 mm) of metastatic lymph node, so called as “micrometastasis,” and selected the largest tumor volume among metastatic lymph nodes in each case. As a control, we conducted Cav-1 immunostaining on 11 normal gastric tissues. Those samples were obtained from resected gastric tissue due to gastric ulcer. Submitting samples to Cav-1 immunostain were far away from the lesion to avoid a significant inflammation. The whole tissue sections of all surgical tumor samples were deparaffinized with xylene and graded alcohols and then rehydrated with distilled water. Endogenous peroxidase was blocked by 3% hydrogen peroxide in methanol for 10 min. Antigen retrieval was then performed by heating the slides for 15 minutes in 0.01 mol/L citrate buffer (pH 6.0). The sections were incubated with human-specific antibodies against caveolin-1 (1 : 400, cell signaling) at room temperature for 2 hours, washed in TBST (tris buffered saline with 0.1% Tween 20), and then incubated with biotinylated secondary antibody for 10 min. After being washed with TBST, sections were stained by a streptavidin-peroxidase detection system (Novex). The immunoreaction was visualized using 3,3′-diaminobenzidine as chromogen and counterstained with hematoxylin. The results were analyzed by two board certified pathologists (S.A.H. and S.H.Y), independently, who were blinded to all patients' clinical data. Expression of primary tumoral, stromal, and metastatic tumoral Cav-1 was analyzed through intensity and proportion. The staining intensity was scored semiquantitively as 0 (negative), +1 (weak), +2 (moderate), and +3 (strong). The H score (0 to 300) was calculated by multiplying intensity and proportion of Cav-1 expression. To find the best cutoff values for predicting survival, a maximally selected rank statistics test was performed, using R Maxstat Package (version 3.3.1; R Foundation for statistical Computing, Viena, Austria) [12]. As a result, H score > 30 for primary tumoral Cav-1 expression, >120 for stromal, and any expression for metastatic tumor were determined as high expression.

### 2.3. Statistical Analysis

Cancer-specific survival (CSS) was calculated from the date of surgery to the date of death from GC; the observations were censored at death from causes other than GC. The relapse-free survival (RFS) duration was calculated from the date of diagnosis to the date of first distant or local disease recurrence or last follow-up. The Kaplan-Meier method was used to analyze “time-to-event” data, and the significance of differences in the cumulative survival curves was evaluated using the log-rank test. Cox proportional hazards regression models were used to investigate the significance of prognostic factors. Cav-1 expression and all variables with a *P* value of <0.25 in the univariate analysis were included in the multivariate analysis. Correlations between immunohistochemical profiles and clinicopathological variables were analyzed by the chi-squared or Fisher's exact test. Comparisons of immunohistochemical expression were performed with an independent-sample *t*-test for continuous variables. Survival rates and hazard ratios (HRs) are presented with their respective 95% confidence intervals (CIs). A *P* value of <0.05 was considered to indicate statistical significance. All statistical analyses were performed using the R statistical software package (R Foundation for Statistical Computing, Vienna, Austria).

## 3. Results

### 3.1. Patients' Clinical Characteristics

In total, 145 paraffin blocks of tumor samples were available from patients who had undergone surgical curative gastrectomy. The clinical and pathological characteristics of the cohort are shown in Table 1. The patient cohort consisted of 107 males (73.8%) and 38 females (26.2%), with a median age of 60 (29–89) years. According to the AJCC staging criteria, twenty patients (13.8%) had stage I disease, 44 (30.3%) patients had stage II disease, and 81 (55.9%) had stage III disease. Ninety-one patients (62.8%) have regional lymph node metastases at the time of operation. Seventy-five (51.7%) patients postoperatively received 5-fluorouracil and cisplatin combination therapy. The follow-up period ranged from 0.7 to 172.2 months, with a median of 70.3 months after curative surgical resection. Of the 145 total patients, 43 (29.7%) died due to their cancer, and 102 (70.3%) were alive at the last follow-up. Disease recurrence was observed in 62 cases (42.8%).

### 3.2. Immunohistochemical Staining Patterns and Relationship with Clinicopathological Findings

**Figure 1:**
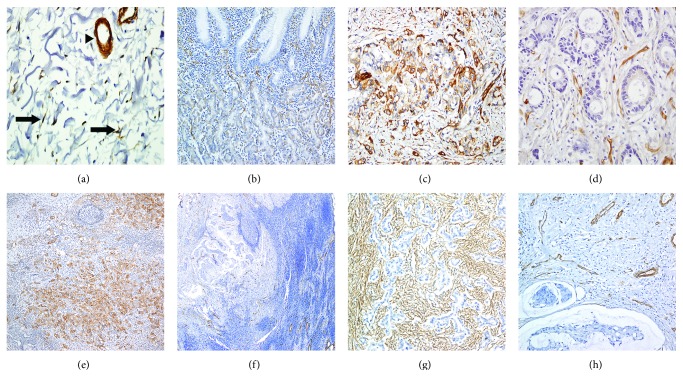
Representative caveolin-1 (Cav-1) expression. In nonneoplastic gastric tissue, Cav-1 expression was detected in fibroblast (arrow) and vessel walls (arrow head) in stroma (a), while Cav-1 showed a scant expression in parietal cells in epithelium (b). Tumor cells showed high (c) and low expression (d) of Cav-1 in the stomach and high (e) and low expression (f) of metastatic lymph node. Stromal Cav-1 immunoreactivity was observed in low (g) and high (h).

**Figure 2:**
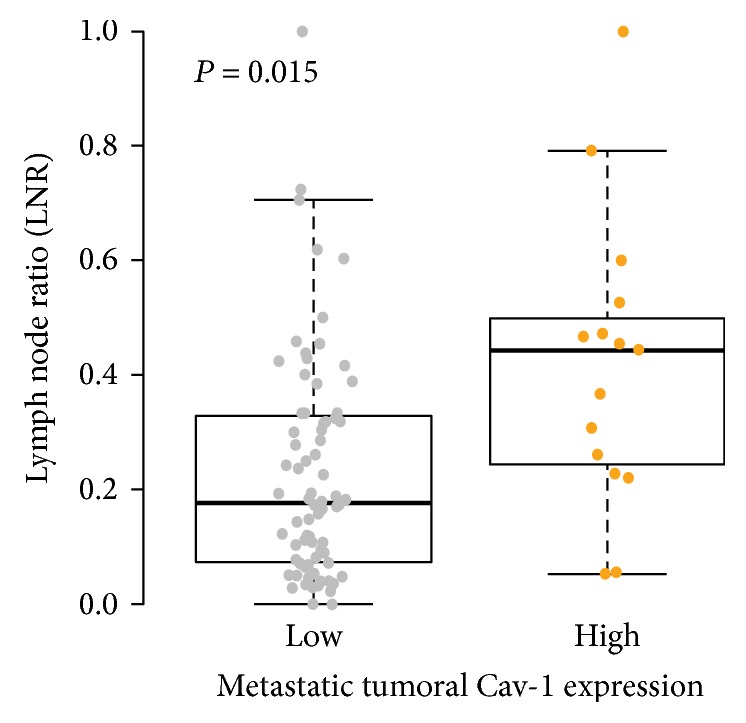
Relationship between Cav-1 protein expression and lymph node ratio (LNR). Patients with a high Cav-1 metastatic tumor showed significantly higher LNR levels (0.229 ± 0.195) compared to those with low metastatic tumoral Cav-1 expression (0.416 ± 0.255, *P* = 0.015).

Figure 1() shows a representative immunohistochemistry results. Cav-1 expression in the normal gastric mucosa was found in parietal cells, but not gastric foveolar epithelium. In stroma, Cav-1 expression was observed in fibroblast, blood vessel, and smooth muscle of muscularis mucosa and proper muscle layer. The result of normal gastric tissue was consistent in all 11 normal gastric tissues (Figures 1(a) and 1(b)). Of the 145 gastric specimens, Cav-1 was highly expressed in tumor cells of 18 (12.4%) cases (Figure 1(c)), while low expression was observed in 127 (87.6%) cases (Figure 1(d)). In a total of 91 cases with nodal metastasis, high expression of Cav-1 of tumor cell in metastatic lymph nodes was observed in 15 (16.5%) cases (Figure 1(e)). In metastatic lymph nodes, 76 (83.5%) cases with low Cav-1 expression were entirely negative for Cav-1 immunostaining (Figure 1(f)). In primary tumoral storma, high expression of Cav-1, which was observed in fibroblast-like cells, was demonstrated in 32 (22.7%) cases (Figure 1(e)), while low expression was found in 109 (77.3%), including loss of stromal Cav-1 expression in 57 (40.4%, Figure 1(f)). Correlation between primary tumoral, stromal, and metastatic tumoral Cav-1 expression was not identified (primary tumor versus metastatic tumor, *P* = 1.000; primary stroma versus metastatic tumor, *P* = 0.522). Associations between Cav-1 expression and clinicopathological features, including well-known prognostic factors such as pathologic TNM stage, lymph node metastasis, lymphovascular invasion, degree of differentiation, and Lauren classification, were also explored. High metastatic tumoral Cav-1 expression was marginally related with vascular invasion (*P* = 0.069), and low expression of Cav-1 in the stroma of the primary tumor was significantly related with diffuse type of Lauren classification (*P* = 0.045, Table 2). The lymph node ratio (LNR), which is defined as the total number of positive/total number of lymph nodes collected, also has been highlighted as an important prognostic indictor of gastric cancer after surgery [13]. Thus, we also investigated the relation between the level of Cav-1 expression and LNR. As shown in Figure 2(), mean LNR level was significantly higher in patients with gastric cancer with high metastatic tumoral Cav-1 expression (0.229 ± 0.195) compared to those with low metastatic tumoral Cav-1 expression (0.416 ± 0.255, *P* = 0.015).

### 3.3. Survival Analysis with Respect to Cav-1 Expression in Primary and Metastatic Lymph Nodes

**Figure 3:**
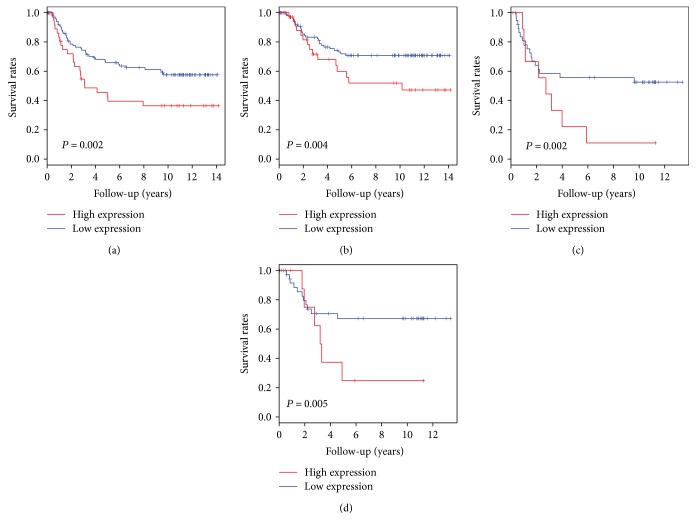
Kaplan-Meier curves for patient survival. Patients with a high Cav-1 metastatic tumor showed a significantly shorter relapse-free survival (a) and cancer-specific survival (b) after surgery than those with a low Cav-1 tumor (*P* = 0.002 and *P* = 0.004, resp.). In patients with lymph node metastasis, high metastatic tumoral Cav-1 expression was associated with shorter relapse-free survival (c) and cancer-specific survival times (d) (*P* = 0.002 and *P* = 0.005, resp.).

The 5-year RFS rate and CSS rate for patients who had undergone curative resection of gastric cancer were 59.7% (95% CI, 51.8–68.7) and 69.9% (95% CI, 62.2–78.6), respectively. For RFS, univariate analysis revealed that the following factors were significantly correlated with disease relapse: advanced pT stage (*P* < 0.001), lymph node metastasis (*P* < 0.001), LNR (*P* < 0.001), lymphatic invasion (*P* = 0.012), and tumor grade (*P* = 0.046) (Table 3). Kaplan-Meier survival curve revealed inverse associations between high expression of metastatic tumoral Cav-1 protein in lymph node and disease relapse (*P* = 0.002), not primary tumoral or stromal Cav-1 expression (*P* = 0.892 and *P* = 0.131, resp., Figure 3(a)). In the multivariate analysis for RFS, in addition to old age, advanced pT stage, and diffuse type, metastatic tumoral Cav-1 expression was an independent indicator of poor prognosis (HR, 3.934; 95% CI, 1.882–8.224; *P* = 0.001, Table 3). For CSS, univariate analysis revealed that the following factors were significantly correlated with CSS: advanced pT stage (*P* = 0.005), lymph node metastasis (*P* < 0.001), LNR (*P* < 0.001), lymphatic invasion (*P* = 0.012), and venous invasion (*P* = 0.023). Kaplan-Meier survival curve revealed a significant association between high expression of tumoral Cav-1 protein in metastatic lymph node and cancer-specific death (*P* = 0.004, Figure 3(b)). In the multivariate analysis, tumoral Cav-1 protein in metastatic lymph node was found to be significantly associated with a poor outcome (HR, 2.681; 95% CI, 1.612–8.623; *P* = 0.002, Table 4). Furthermore, to clarify the role of Cav-1 expression in the patients with lymph node metastasis, we performed univariate and multivariate analyses for RFS and CSS in the subgroup of the patients with lymph node metastasis. Metastatic tumoral Cav-1 expression remained as a strong indicator of poor prognosis for RFS (HR, 3.136; 95% CI, 1.444–6.810; *P* = 0.003, Figure 3(c) and Table 5) and CSS (HR, 2.509; 95% CI, 1.078–5.837; *P* = 0.032, Figure 3(d) and Table 6).

## 4. Discussion

The impact of Cav-1 on cancer progression, whether it is expressed in the tumor cells or stromal cells, seems to be complex and debatable [5, 8, 14–17]. Previous reports also showed a controversial role for Cav-1 expression in GC, leading us to analyze Cav-1 expression in GC [10, 15, 18]. In the present study, we found that Cav-1 expression was more frequently observed in tumor cells of metastatic lymph nodes than of primary sites and that high metastatic tumoral Cav-1 expression was associated with poor clinical outcome in the patients with resected GC, not primary tumoral or stromal Cav-1 expression. Its prognostic influence seemed to be independent of well-known clinicopathological factors. To the best of our knowledge, this is the first report focusing on clinical significance of Cav-1 in metastatic tumors in the patients with resected GC.

Cav-1 protein is a component of caveolae invaginated microdomains of the plasma membrane that is present in most mammalian cells [5]. High tumoral Cav-1 expression was observed in GC cells in 18 of 145 cases (12.4%). In the nonepithelial compartment, Cav-1 was more frequently expressed in fibroblast-like cells, endothelial cells, and smooth muscle within the stroma surrounding tumor nests more than tumor cell, and loss of stromal Cav-1 expression was found in 57 of 141 cases (40.4%), which is consistent with the previous studies [10, 18]. In a study of 405 patients with GC, Cav-1 expression was absent in normal epithelial cells and was highly expressed in 22 (5.4%) of 405 cases [10]. In addition, loss of stromal Cav-1 expression in cancer-associated fibroblast (CAF) was reported at 35% in GC [18]. Similarly, Cav-1 protein was dysregulated in several other gastrointestinal cancers. Tumoral Cav-1 expression increased in cancers cells compared to their normal counterparts, whereas stromal Cav-1 expression decreased in cancer tissues compared to adjacent normal tissues, such as pancreatic cancer [9, 19], esophageal cancer [20], and hepatocellular carcinomas [21]. Cav-1 expression is known to be regulated mainly by inactivation of oncogenes or inactivation of tumor suppressive genes, TGF-*β*, and oxidative stress in the tumor microenvironment [22, 23].

The role of Cav-1 protein in tumorigenesis and tumor progression still remains controversial. Initially, Cav-1 protein has been shown to play a role in tumor suppression. The *CAV-1* gene resides on chromosome 7q31.1, a fragile site known as FRA7G, which is commonly deleted in human cancers [24]. Mutant mice in the gene-encoding *CAV-1* showed an enhanced association with the development and progression of breast cancer carcinogenesis [6] and normally regulated the proliferation of intestinal stem cells in vivo [14]. Tumor suppressive function of Cav-1 is mediated by induction of the cell cycle arrest of G0-G1-phase in fibroblasts through a p53/p21-dependent pathway [25], inhibition of Wnt/*β*-catenin signaling in epithelial cell [26], and activation of cell-matrix interactions toward the basal membrane [27]. However, the upregulation of Cav-1 in human cancer cells may serve as a tumor promoter role in the majority of human cancer types. Previous studies have related Cav-1 overexpression with oncogenic transformation, invasion, and metastasis. Recently, Chatterjee et al. have observed that *CAV-1* knockdown reduced proliferative, invasive, and migratory properties in multiple pancreatic cancer cell lines [9]. Cav-1 affects several signaling pathways in cellular transformation, including aerobic glycolysis, JAK/STAT, JNK, and Src signaling pathway [9, 28]. These conflicting effects may be mediated by the activation status of different domains of Cav-1, which depends on the levels of other molecules in different signaling pathways that are expressed with Cav-1 [11].

Notably, in a study of GC, Cav-1 mRNA expression was lower in cell lines derived from a primary tumor, but it increased in cell lines originating from distant metastases. In addition, Cav-1 overexpressing gastric cancer cell line gained prosurvival ability [15], which results are concordant with ours that the high Cav-1 expression of tumor cells in metastatic lymph node was related with poor prognosis of GC patients. Although primary tumoral Cav-1 overexpression was documented to associate with lymph node metastasis and advanced TNM stage in the previous study [10], our results suggested that primary tumoral Cav-1 expression was not correlated with these clinicopathological features and clinical outcomes, which is in consistency with Barresi et al.'s study [29]. However, our present study showed that metastatic tumoral Cav-1 expression was related with high LNR (*P* = 0.015) and perivascular invasion (*P* = 0.069) and that its elevated expression had a strongly negative correlation with clinical prognosis, which suggest that Cav-1 protein plays a more significant role in metastatic tumor than the primary tumor. Ectopic expression of Cav-1 in GC cell lines with a low level of Cav-1 decreased proliferation but promoted anchorage-independent growth and survival. This biphasic pattern may support the different roles of Cav-1 as a tissue and stage-specific tumor modulator [5], where it acts as an inhibitor or promoter of tumor formation and progression depending on its protein interaction partners such as growth factor receptors or cell adhesion molecules.

In this study, regarding stromal Cav-1 expression, it was more frequently downregulated in diffuse-type (58.2%) than mixed or intestinal-type (41.8%) of Lauren classification (*P* = 0.045), which is consistent with the previous reports [29, 30], and stromal Cav-1 protein did not show the prognostic role in curatively resected GC. The downregulation of Cav-1 is a major characteristic of CAFs, and existing studies have indicated that CAFs have the ability to inhibit cancer cell apoptosis, increase the growth of cancer cells, and activate tumor angiogenesis [16, 18]. Thus, the loss of stromal Cav-1 has been reported to be a poor prognostic factor in various human cancers [31]. In previous study, low expression of Cav-1 in CAF was related with unfavorable prognosis in GC [30]. Compared to the present study including only primary R0 resection, the patient cohort in that study was heterogeneous, in which patients who received cytoreductive surgery and postoperative radiotherapy were included. Moreover, we used whole tissue section to evaluate the expression of Cav-1, while they conducted only on tissue microarray. These factors might be contributed to the different results of prognostic impact of stromal Cav-1 expression in GC. Furthermore, the prognostic significance of Cav-1 in CAFs remains still debatable in various cancers. Goetz et al. reported that Cav-1 expression in the CAFs of breast cancer correlated with low survival and that stromal Cav-1, through p190RhoGAP regulation, favors remodels peri- and intratumoral microenvironments to facilitate tumor invasion, correlating with increased metastatic potency in vitro and in vivo [16]. Vered et al., in an analysis of their series of 64 cases of tongue squamous cell carcinoma, reported that accumulation of Cav-1 in tissue microenvironment had a negative prognostic value and also showed that Cav-1 expression in fibroblasts undergoes transdifferentiation to CAFs [17]. These conflicting results may attribute to the contradictory function of CAF in cancer progression [32].

The limitation of our study is that Cav-1 expression was evaluated in a part of metastatic lymph nodes. Thus, the issue was remained to represent metastatic gastric cancer. However, we excluded meticulously low tumor volume of metastatic lymph node (<2 mm), so called as “micrometastasis,” and selected the largest tumor volume among the metastatic lymph nodes in each case. We believe that the metastatic lymph node in the present study could be sufficient to evaluate Cav-1 expression.

This study clearly demonstrates the complex role of Cav-1 in GC, of which metastatic tumoral expression was an independent prognostic factor for clinical outcomes, not primary tumoral expression of Cav-1. Future study will be required to determine the mechanism of Cav-1 protein, by which oncogenic signaling through this complex is regulated.

## Figures and Tables

**Table 1:** Baseline clinicopathological characteristics of patients with gastric cancer.

Characteristics	No. of patients, *n* (%)
Patient number	145
Age (years), median	60 (29–89)
>60	73 (50.3)
≤60	72 (49.7)
Gender	
Male	107 (73.8)
Female	38 (26.2)
T stage	
pT2-3	43 (29.7)
pT4	102 (70.3)
N stage	
pN0-1	79 (54.5)
pN2-3	66 (45.5)
TNM stage	
I	20 (13.8)
II	44 (30.3)
III	81 (55.9)
Tumor grade	
Well	16 (11.0)
Moderate–poor	129 (89.0)
Lymphatic invasion	
No	27 (18.8)
Yes	117 (81.2)
Vascular invasion	
No	127 (91.4)
Yes	12 (8.6)
Lauren classification	
Nondiffuse type	68 (46.9)
Diffuse type	77 (53.1)
LNR, median (range)	0.071 (0-1)
>median	74 (51.0)
≤median	71 (49.0)
Adjuvant therapy	
No	70 (48.3)
Yes	75 (51.7)

LNR: lymph node ratio.

**Table 2:** Correlations between clinicopathologic findings and caveolin-1 expression.

	Primary tumoral Cav-1 expression		Stromal Cav-1 expression		Metastatic tumoral Cav-1 expression	
	Low, *n* (%)	High, *n* (%)	Low, *n* (%)	High, *n* (%)	Low, *n* (%)	High, *n* (%)
No. of patients	127 (87.6)	18 (12.4)	109 (77.3)	32 (22.7)	76 (83.5)	15 (16.5)
TNM stage						
I	16 (12.6)	4 (22.2)	16 (14.7)	4 (12.5)	1 (1.3)	0 (0)
II	38 (29.9)	6 (33.3)	33 (30.3)	9 (28.1)	10 (13.2)	3 (20.0)
III	73 (57.5)	8 (44.5)	60 (55.0)	19 (59.4)	65 (85.5)	12 (80.0)
*P* value		0.431		0.960		0.540
Tumor grade						
Well	15 (11.8)	1 (5.6)	9 (8.3)	6 (18.8)	3 (3.9)	1 (6.7)
Moderately–poorly	112 (88.2)	17 (94.4)	100 (91.7)	26 (81.2)	73 (96.1)	14 (93.3)
*P* value		0.694		0.106		0.520
Lymphatic invasion						
No	22 (17.5)	5 (27.8)	21 (19.4)	6 (18.8)	4 (5.3)	0 (0)
Yes	104 (82.5)	13 (72.2)	87 (80.6)	26 (81.2)	72 (94.7)	15 (100)
*P* value		0.333		1		1
Vascular invasion						
No	110 (90.9)	17 (94.4)	95 (92.2)	28 (87.5)	67 (90.3)	10 (71.4)
Yes	11 (9.1)	1 (5.6)	8 (7.8)	4 (12.5)	7 (9.70)	4 (28.6)
*P* value		1		0.478		0.069
Lauren classification						
Nondiffuse type	60 (47.2)	8 (44.4)	46 (41.8)	20 (62.5)	29 (37.8)	9 (60.0)
Diffuse type	67 (52.8)	10 (55.6)	63 (58.2)	12 (37.5)	47 (62.2)	6 (40.0)
*P* value		1		0.045^∗^		0.154

^∗^Statistically significant (*P* < 0.05).

**Table 3:** Univariate and multivariate analyses of relapse-free survival rates using the Cox proportional hazards model in all patients.

Characteristics	Univariate analysis			Multivariate analysis		
	Hazard ratio	95% CI	*P* value	Hazard ratio	95% CI	*P* value
Age (>60 versus ≤60 years)	1.623	0.982–2.683	0.059	2.158	1.207–3.862	0.009
Sex (female versus male)	0.804	0.45–1.439	0.463			
Advanced T stage (pT4 versus pT2-3)	3.088	1.557–6.045	0.001	2.464	0.995–6.102	0.051
Advanced N stage (pN2-3 versus pN0-1)	3.688	2.157–6.304	<0.001	1.491	0.208–2.156	0.502
Lauren classification (diffuse versus nondiffuse)	1.505	0.905–2.501	0.115	1.917	1.032–3.562	0.039
Lymphatic invasion (yes versus no)	2.956	1.272–6.869	0.012	1.479	0.543–0.032	0.444
Venous invasion (yes versus no)	1.821	0.782–4.238	0.165	0.483	0.778–5.498	0.145
Tumor grade (moderate–poor versus well)	4.199	1.026–17.184	0.046	1.754	0.112–2.911	0.498
Adjuvant chemotherapy (yes versus no)	0.839	0.51–1.381	0.490			
LNR (>median versus ≤median)	4.551	2.567–8.069	<0.001	2.282	0.926–5.626	0.072
Tumoral Cav-1 expression, primary tumor (high versus low)	0.95	0.452–1.996	0.892	1.275	0.485–3.350	0.622
Stromal Cav-1 expression, primary tumor (high versus low)	1.529	0.881–2.652	0.131	0.956	0.516–2.116	0.902
Tumoral Cav-1 expression, lymph node (high versus low)	2.874	1.491–5.540	0.002	3.934	1.882–8.224	<0.001

LNR: lymph node ratio.

**Table 4:** Univariate and multivariate analyses by cancer-specific survival rates using the Cox proportional hazards model in all patients.

Characteristics	Univariate analysis			Multivariate analysis		
	Hazard ratio	95% CI	*P* value	Hazard ratio	95% CI	*P* value
Age (>60 versus ≤60)	1.143	0.623–2.095	0.666			
Sex (female versus male)	0.9	0.452–1.792	0.765			
Advanced T stage (pT4 versus pT2-3)	3.452	1.453–8.202	0.005	3.366	1.010–11.214	0.048
Advanced N stage (pN2-3 versus pN0-1)	4.344	2.214–8.522	<0.001	1.919	0.147–1.838	0.310
Lauren classification (diffuse versus nondiffuse)	1.532	0.827–2.838	0.175	0.506	0.928–4.204	0.077
Lymphatic invasion (yes versus no)	12.903	1.774–93.854	0.012	5.759	0.718–46.228	0.099
Venous invasion (yes versus no)	2.744	1.149–6.553	0.023	0.380	0.990–6.990	0.052
Tumor grade (moderate–poor versus well)	2.866	0.692–11.86	0.146	2.983	0.057–1.970	0.226
Adjuvant chemotherapy (yes versus no)	0.794	0.433–1.454	0.454			
LNR (>median versus ≤median)	6.476	2.987–14.042	<0.001	0.2662	1.122–12.579	0.031
Tumoral Cav-1 expression, primary tumor (high versus low)	0.496	0.153–1.605	0.242	0.3945	0.084–1.849	0.237
Stromal Cav-1 expression, primary tumor (high versus low)	1.48	0.756–2.897	0.253			
Tumoral Cav-1 expression, lymph node (high versus low)	3.064	1.429–6.569	0.004	2.681	1.612–8.623	0.002

LNR: lymph node ratio.

**Table 5:** Univariate and multivariate analyses of relapse-free survival rates using the Cox proportional hazards model in patients with metastatic lymph nodes.

Characteristics	Univariate analysis			Multivariate analysis		
	Hazard ratio	95% CI	*P* value	Hazard ratio	95% CI	*P* value
Age (>60 versus ≤60 years)	2.254	1.295–3.922	0.004	2.252	1.251–4.052	0.006
Sex (female versus male)	1.331	0.722–2.453	0.360			
Advanced T stage (pT4 versus pT2-3)	2.414	1.030–5.655	0.042	2.750	1.110–6.810	0.028
Advanced N stage (pN2-3 versus pN1)	2.064	1.059–4.022	0.033	1.767	0.535–5.834	0.350
Lauren classification (diffuse versus nondiffuse)	1.411	0.809–2.462	0.225			
Lymphatic invasion (yes versus no)	1.103	0.344–3.542	0.869			
Venous invasion (yes versus no)	1.512	0.644–3.552	0.343			
Tumor grade (moderate–poor versus well)	1.429	0.348–5.876	0.620			
Adjuvant chemotherapy (yes versus no)	0.450	0.262–0.775	0.004	0.517	0.266–1.002	0.050
LNR (>median versus ≤median)	2.910	1.309–6.468	0.009	2.154	0.873–5.315	0.095
Tumoral Cav-1 expression, primary tumor (high versus low)	1.090	0.466–2.551	0.842	0.793	0.264–2.383	0.680
Stromal Cav-1 expression, primary tumor (high versus low)	1.354	0.751–2.441	0.313	1.122	0.575–2.186	0.735
Tumoral Cav-1 expression, lymph node (high versus low)	2.79	1.447–5.379	0.002	3.136	1.444–6.810	0.003

LNR: lymph node ratio.

**Table 6:** Univariate and multivariate analyses of cancer-specific survival rates using the Cox proportional hazards model in patients with metastatic lymph nodes.

Characteristics	Univariate analysis			Multivariate analysis		
	Hazard ratio	95% CI	*P* value	Hazard ratio	95% CI	*P* value
Age (>60 versus ≤60 years)	1.723	0.901–3.300	0.100	2.248	1.023–4.937	0.437
Sex (female versus male)	1.524	0.751–3.091	0.243			
Advanced T stage (pT4 versus pT2-3)	3.318	1.018–10.816	0.047	3.905	1.173–12.992	0.026
Advanced N stage (pN2-3 versus pN1)	2.379	1.041–5.436	0.040	2.100	0.635–8.025	0.252
Lauren classification (diffuse versus nondiffuse)	1.291	0.669–2.491	0.446			
Lymphatic invasion (yes versus no)	2.352	0.322–17.163	0.399			
Venous invasion (yes versus no)	2.233	0.923–5.399	0.075	1.848	0.709–4.818	0.208
Tumor grade (moderate–poor versus well)	1.083	0.260–4.505	0.913			
Adjuvant chemotherapy (yes versus no)	0.487	0.255–0.932	0.030	3.905	1.173–12.992	0.032
LNR (>median versus ≤median)	4.911	1.505–16.03	0.008	3.451	1.026–11.606	0.045
Tumoral Cav-1 expression, primary tumor (high versus low)	0.509	0.122–2.116	0.353	0.328	0.071–1.512	0.152
Stromal Cav-1 expression, primary tumor (high versus low)	1.324	0.652–2.687	0.437			
Tumoral Cav-1 expression, lymph node (high versus low)	2.985	1.392–6.399	0.005	2.509	1.078–5.837	0.032

LNR: lymph node ratio.

## References

[1] Torre L. A., Bray F., Siegel R. L., Ferlay J., Lortet-Tieulent J., Jemal A. (2015). Global cancer statistics, 2012. *CA: A Cancer Journal for Clinicians*.

[2] Bang Y. J., Cutsem E. V., Feyereislova A. (2010). Trastuzumab in combination with chemotherapy versus chemotherapy alone for treatment of HER2-positive advanced gastric or gastro-oesophageal junction cancer (ToGA): a phase 3, open-label, randomised controlled trial. *Lancet*.

[3] Fuchs C. S., Tomasek J., Yong C. J. (2014). Ramucirumab monotherapy for previously treated advanced gastric or gastro-oesophageal junction adenocarcinoma (REGARD): an international, randomised, multicentre, placebo-controlled, phase 3 trial. *Lancet*.

[4] Cohen A. W., Hnasko R., Schubert W., Lisanti M. P. (2004). Role of caveolae and caveolins in health and disease. *Physiological Reviews*.

[5] Williams T. M., Lisanti M. P. (2005). Caveolin-1 in oncogenic transformation, cancer, and metastasis. *American Journal of Physiology Cell Physiology*.

[6] Hnasko R., Lisanti M. P. (2003). The biology of caveolae: lessons from caveolin knockout mice and implications for human disease. *Molecular Interventions*.

[7] Bender F. C., Reymond M. A., Bron C., Quest A. F. (2000). Caveolin-1 levels are down-regulated in human colon tumors, and ectopic expression of caveolin-1 in colon carcinoma cell lines reduces cell tumorigenicity. *Cancer Research*.

[8] Wiechen K., Sers C., Agoulnik A. (2001). Down-regulation of caveolin-1, a candidate tumor suppressor gene, in sarcomas. *The American Journal of Pathology*.

[9] Chatterjee M., Ben-Josef E., Thomas D. G. (2015). Caveolin-1 is associated with tumor progression and confers a multi-modality resistance phenotype in pancreatic cancer. *Scientific Reports*.

[10] Nam K. H., Lee B. L., Park J. H. (2013). Caveolin 1 expression correlates with poor prognosis and focal adhesion kinase expression in gastric cancer. *Pathobiology*.

[11] Fine S. W., Lisanti M. P., Galbiati F., Li M. (2001). Elevated expression of caveolin-1 in adenocarcinoma of the colon. *American Journal of Clinical Pathology*.

[12] Tejero R., Navarro A., Campayo M. (2014). miR-141 and miR-200c as markers of overall survival in early stage non-small cell lung cancer adenocarcinoma. *PloS One*.

[13] Bando E., Yonemura Y., Taniguchi K., Fushida S., Fujimura T., Miwa K. (2002). Outcome of ratio of lymph node metastasis in gastric carcinoma. *Annals of Surgical Oncology*.

[14] Li J., Hassan G. S., Williams T. M. (2005). Loss of caveolin-1 causes the hyper-proliferation of intestinal crypt stem cells, with increased sensitivity to whole body gamma-radiation. *Cell Cycle*.

[15] Burgermeister E., Xing X., Rocken C. (2007). Differential expression and function of caveolin-1 in human gastric cancer progression. *Cancer Research*.

[16] Goetz J. G., Minguet S., Navarro-Lerida I. (2011). Biomechanical remodeling of the microenvironment by stromal caveolin-1 favors tumor invasion and metastasis. *Cell*.

[17] Vered M., Lehtonen M., Hotakainen L. (2015). Caveolin-1 accumulation in the tongue cancer tumor microenvironment is significantly associated with poor prognosis: an in-vivo and in-vitro study. *BMC Cancer*.

[18] Shen X. J., Zhang H., Tang G. S. (2015). Caveolin-1 is a modulator of fibroblast activation and a potential biomarker for gastric cancer. *International Journal of Biological Sciences*.

[19] Suzuoki M., Miyamoto M., Kato K. (2002). Impact of caveolin-1 expression on prognosis of pancreatic ductal adenocarcinoma. *British Journal of Cancer*.

[20] Kato K., Hida Y., Miyamoto M. (2002). Overexpression of caveolin-1 in esophageal squamous cell carcinoma correlates with lymph node metastasis and pathologic stage. *Cancer*.

[21] Tang Y., Zeng X., He F., Liao Y., Qian N., Toi M. (2012). Caveolin-1 is related to invasion, survival, and poor prognosis in hepatocellular cancer. *Medical Oncology*.

[22] Witkiewicz A. K., Kline J., Queenan M. (2011). Molecular profiling of a lethal tumor microenvironment, as defined by stromal caveolin-1 status in breast cancers. *Cell Cycle*.

[23] Bist A., Fielding C. J., Fielding P. E. (2000). p53 regulates caveolin gene transcription, cell cholesterol, and growth by a novel mechanism. *Biochemistry*.

[24] Engelman J. A., Zhang X. L., Lisanti M. P. (1998). Genes encoding human caveolin-1 and -2 are co-localized to the D7S522 locus (7q31.1), a known fragile site (FRA7G) that is frequently deleted in human cancers. *FEBS Letters*.

[25] Galbiati F., Volonte D., Liu J. (2001). Caveolin-1 expression negatively regulates cell cycle progression by inducing G(0)/G(1) arrest via a p53/p21(WAF1/Cip1)-dependent mechanism. *Molecular Biology of the Cell*.

[26] Sotgia F., Williams T. M., Cohen A. W., Minetti C., Pestell R. G., Lisanti M. P. (2005). Caveolin-1-deficient mice have an increased mammary stem cell population with upregulation of Wnt/beta-catenin signaling. *Cell Cycle*.

[27] Wary K. K., Mariotti A., Zurzolo C., Giancotti F. G. (1998). A requirement for caveolin-1 and associated kinase Fyn in integrin signaling and anchorage-dependent cell growth. *Cell*.

[28] Ha T. K., Her N. G., Lee M. G. (2012). Caveolin-1 increases aerobic glycolysis in colorectal cancers by stimulating HMGA1-mediated GLUT3 transcription. *Cancer Research*.

[29] Barresi V., Giuffre G., Vitarelli E., Todaro P., Tuccari G. (2008). Caveolin-1 immuno-expression in human gastric cancer: histopathogenetic hypotheses. *Virchows Archiv*.

[30] Zhao X., He Y., Gao J. (2013). Caveolin-1 expression level in cancer associated fibroblasts predicts outcome in gastric cancer. *PLoS One*.

[31] Chen D., Che G. (2014). Value of caveolin-1 in cancer progression and prognosis: emphasis on cancer-associated fibroblasts, human cancer cells and mechanism of caveolin-1 expression (Review). *Oncology Letters*.

[32] Kalluri R., Zeisberg M. (2006). Fibroblasts in cancer. *Nature Reviews Cancer*.

